# Obstructive sleep apnea and comorbidities: a dangerous liaison

**DOI:** 10.1186/s40248-019-0172-9

**Published:** 2019-02-14

**Authors:** Maria R. Bonsignore, Pierpaolo Baiamonte, Emilia Mazzuca, Alessandra Castrogiovanni, Oreste Marrone

**Affiliations:** 1 Division of Respiratory Medicine, Biomedical Department of Internal Medicine and Medical Specialties (Di.Bi.M.I.S), University Hospital Paolo Giaccone, University of Palermo, Piazza delle Cliniche, 2, 90100 Palermo, Italy; 2 National Research Council (CNR), Institute of Biomedicine and Molecular Immunology (IBIM), Palermo, Italy; 3 Clinic for Pneumology und Allergology, Center of Sleep Medicine and Respiratory Care, Bethanien Hospital, Solingen, Germany

**Keywords:** Mortality, prognosis, cardiovascular disease, diabetes, asthma, COPD, cancer

## Abstract

Obstructive sleep apnea (OSA) is a highly prevalent disease, and is traditionally associated with increased cardiovascular risk. The role of comorbidities in OSA patients has emerged recently, and new conditions significantly associated with OSA are increasingly reported. A high comorbidity burden worsens prognosis, but some data suggest that CPAP might be protective especially in patients with comorbidities. Aim of this narrative review is to provide an update on recent studies, with special attention to cardiovascular and cerebrovascular comorbidities, the metabolic syndrome and type 2 diabetes, asthma, COPD and cancer. Better phenotypic characterization of OSA patients, including comorbidities, will help to provide better individualized care. The unsatisfactory adherence to CPAP in patients without daytime sleepiness should prompt clinicians to examine the overall risk profile of each patient in order to identify subjects at high risk for worse prognosis and provide the optimal treatment not only for OSA, but also for comorbidities.

Obstructive sleep apnea (OSA) is highly prevalent in the general population, and occurs at all ages [1]. OSA is characterized by collapse of upper airways during sleep with ineffective respiratory efforts, intermittent hypoxia and sleep disruption. Continuous positive airway pressure (CPAP), mandibular advancement devices, and upper airway (UA) and maxillo-facial surgery are therapeutic options that prevent UA closure during sleep, CPAP being the gold standard for moderate-severe OSA. The typical OSA patient is overweight or obese, sleepy in passive situations or while driving, and often affected by systemic hypertension, type 2 diabetes, and dyslipidemia [1].

The frequent association of OSA with metabolic and cardiovascular diseases has been recognized since the early studies, but the role of OSA as an independent risk factor has long remained controversial due to the presence of powerful confounders, such as hypertension and obesity [2]. Interest in the role of comorbidities in OSA has grown in the last decade, as shown by the rising number of publications on the topic (Fig. 1). This review will examine some epidemiological aspects of comorbidities in OSA, and summarizes the current state of the art on the most frequent comorbidities encountered in clinical practice in OSA patients.

**Fig. 1:**
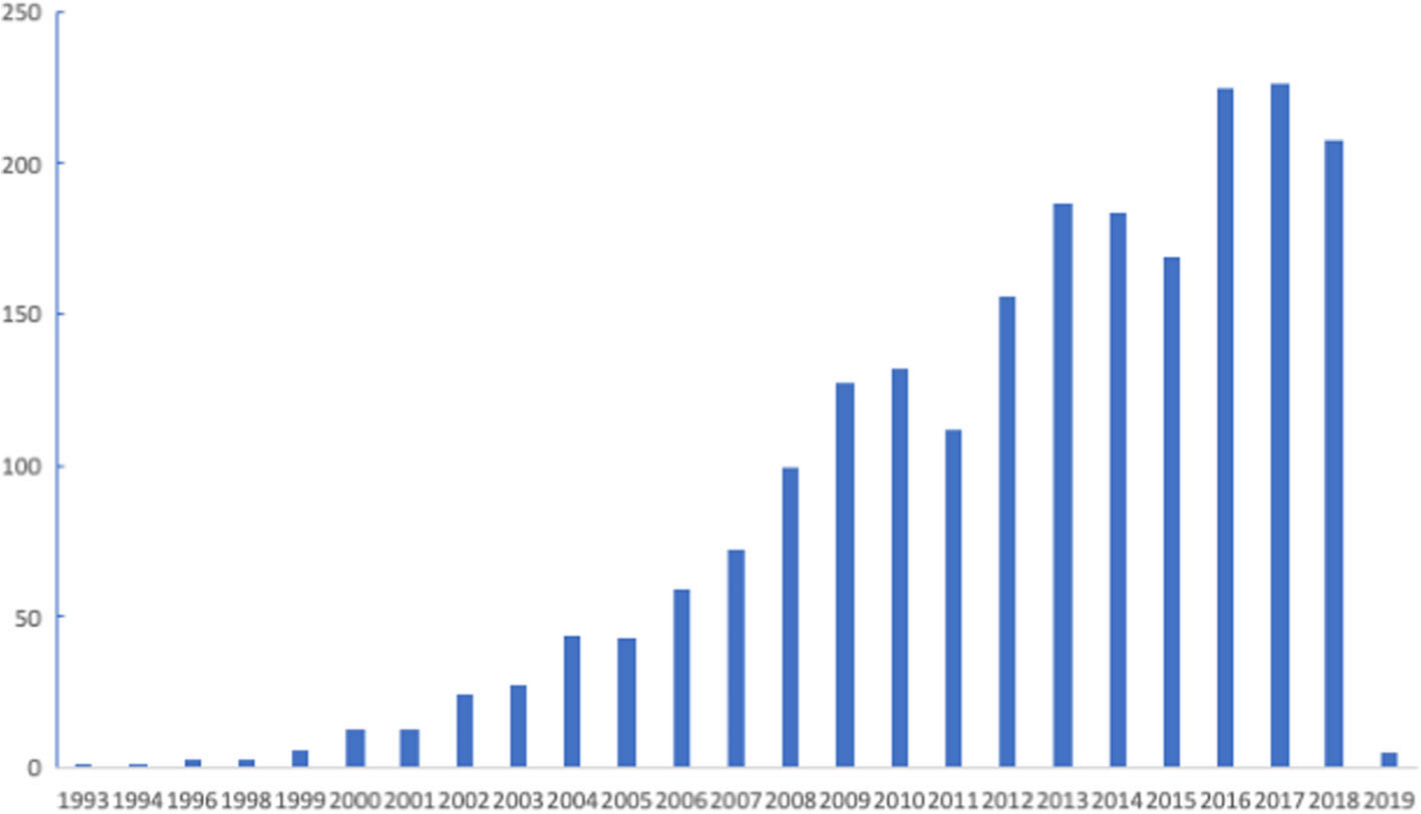
Retrieval of references by searching PubMed for “obstructive sleep apnea” and comorbidities, 9 Jan 2019

## Comorbidities in OSA: the size of the problem

**Fig. 2:**
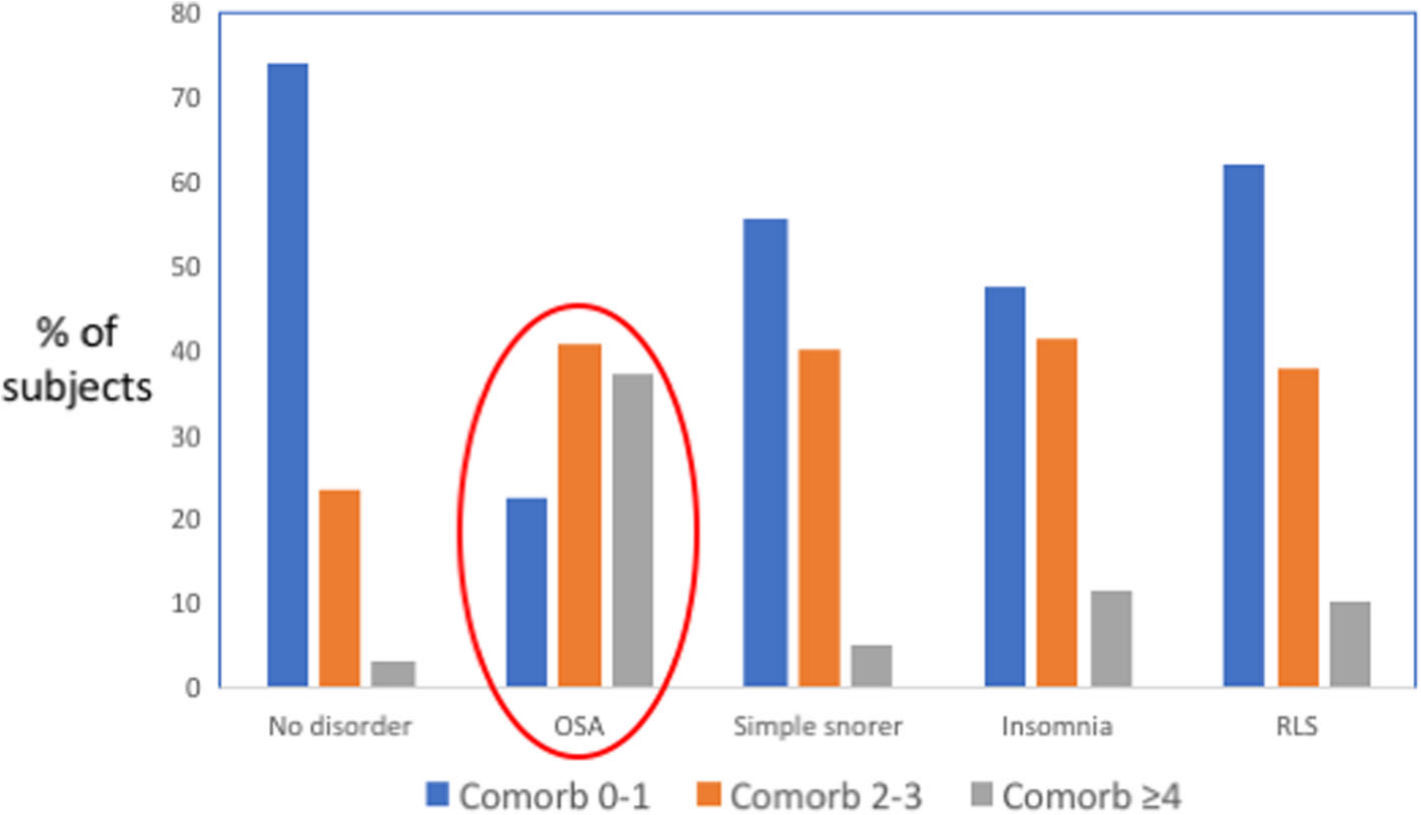
Differently from other common sleep disorders, 80% of patients with obstructive sleep apnea (OSA) show multiple comorbidities. RLS: restless leg syndrome. Drawn based on data from [5]

Currently, comorbidities are a major topic in clinical research on OSA. Several recent studies reported a high prevalence of comorbidities in OSA patients [3–6] (Fig. 2). The distribution of comorbidities differed between men and women, with diabetes and ischemic heart disease being more prevalent in men with OSA, and hypertension and depression being more prevalent in women with OSA compared to non-OSA subjects [3, 7]. According to some studies, the comorbidity burden progressively increases with OSA severity [5, 6, 8, 9].

A recent study from Taiwan in a large number of OSA patients analyzed prevalence of comorbidities at diagnosis and their relationship with mortality risk during follow up [10]. The study confirmed that OSA patients show a high prevalence of cardiovascular diseases (systemic hypertension, coronary artery disease, arrhythmias, ischemic stroke), respiratory diseases (COPD, asthma), and metabolic disorders (diabetes mellitus, dyslipidemia, gout). Many other disorders were also identified, including peptic ulcer disease, gastroesophageal reflux, chronic liver disease, anxiety, insomnia, and depression. The authors identified ten comorbid conditions associated with increased mortality risk, and developed a comorbidity score for OSA by taking into account the relative risk associated with each disease state and the number of comorbidities. Such an approach allows to focus on those comorbidities that are prognostically more relevant in OSA. For example, the highest risk was associated with end-stage renal disease and aortic aneurysm, which showed the lowest prevalence in the sample [10]. In addition, the higher the comorbidity score, the higher the mortality risk [10]. Unfortunately, the impact of OSA treatment was not evaluated in detail.

## Comorbidities and mortality in CPAP-treated OSA

Other studies on the prognostic impact of comorbidities in OSA patients examined the effects of CPAP treatment. In a large study from Denmark, negative predictors for survival were male gender, age ≥60 years, no CPAP treatment, prior comorbidity, and low educational level [11]. Another study found that age and occurrence of comorbidities predicted mortality in OSA patients [12]. In patients aged >50 years, protective effects of CPAP treatment were shown only in patients with comorbidities [12]. In patients with moderate-severe obesity and OSA, treatment with CPAP or noninvasive ventilation was associated with fewer cardiovascular events only in patients with a high number of comorbidities [13]. The protective effect of CPAP might be larger in males than females with OSA [14, 15]. Other observational studies reported a protective effect of CPAP treatment in elderly OSA patients, who usually show a high prevalence of cardiometabolic comorbidities [16, 17].

Therefore, occurrence of comorbidities could identify subgroups of OSA patients at high risk, who might show benefit from CPAP treatment. Several studies have tried to define clinical phenotypes of OSA, and a cluster of patients with few OSA symptoms but high comorbidity burden has been reported by most studies published so far; such a cluster at least partly overlaps with the cluster of elderly OSA patients [18]. More recent analyses pointed to sleep fragmentation and hypoxia as risk factors for cardiovascular events or death, and regular CPAP use appeared to exert a protective effect [19].

## Common comorbidities in OSA patients

A comprehensive review of all possible comorbidities associated with OSA is beyond the scope of this article. Only the most frequent diseases will be discussed, with special attention to the most recent publications.

### Cardiovascular and cerebrovascular diseases

Many studies have examined the role of OSA as a pathogenetic factor in cardiovascular and cerebrovascular diseases, as well as the potential protective effects of CPAP treatment. OSA may increase cardiovascular risk through multiple intermediate mechanisms, such as intermittent hypoxia, high sympathetic nervous activity, systemic hypertension, endothelial cell dysfunction, oxidative stress, inflammation, and accelerated atherosclerosis [1]. On the other hand, chronic intermittent hypoxia could also activate some protective mechanisms, for example through the development of coronary vessel collaterals in patients with ischemic heart disease [20, 21].

#### Systemic hypertension

The best studied cardiovascular comorbidity in OSA is systemic hypertension [22]. Respiratory events during sleep are associated with hypertensive peaks occurring at the end of apneas and hypopneas, increased mean nocturnal blood pressure, and increased variability of blood pressure [23]. A dose-response relationship has been shown between OSA severity and blood pressure [24]. OSA patients may show elevated blood pressure values during sleep only, or during sleep and wakefulness, making 24-hour monitoring of blood pressure highly advisable in the OSA population [22]. Resistant hypertension, i.e. incomplete blood pressure control on three antihypertensive drugs, is also frequent in OSA patients.

Several studies assessed the potential benefit of CPAP treatment on blood pressure values, and meta-analyses demonstrated that on average blood pressure decreased by only a small amount during CPAP treatment. However, the therapeutic effect of CPAP on hypertension varied according to OSA severity, compliance to CPAP treatment, and baseline blood pressure values [25]. OSA patients with resistant hypertension showed a quite large decrease in blood pressure during CPAP [26]. Antihypertensive drugs, and diuretics in particular, may slightly decrease AHI in OSA [27]. In general, pharmacological treatment to control hypertension is necessary in hypertensive OSA patients, given the small effects of CPAP on blood pressure [28, 29].

#### Cardiovascular events and/or death

Several studies have addressed the question of OSA and cardiovascular morbidity and mortality. The prospective cohort study by Marin and coworkers reported a high cardiovascular risk in patients with severe OSA, which was normalized by CPAP treatment [30]. The results of observational studies confirmed the association of untreated OSA with overall and cardiovascular mortality [31]. In patients undergoing percutaneous coronary intervention, OSA was associated with occurrence of cardiovascular events during follow up [32, 33].

Randomized controlled trials (RCTs) in OSA patients with known coronary artery or cerebrovascular disease were then designed to verify whether treatment of OSA in patients at high cardiovascular risk might exert a protective role. However, RCTs on the effects of CPAP in patients with known coronary or cerebrovascular disease failed to show any protective effect of CPAP treatment on cardiovascular risk [34]. Current uncertainty is due to the discrepancy between data obtained from observational studies and RCTs [35]. Different patients’ characteristics according to the type of study may explain the different results. In particular, only patients without excessive daytime sleepiness were included in long-term RCTs, since it would be unethical to withdraw treatment in symptomatic patients. It is known that compliance to CPAP treatment in non-sleepy patients is low, as underlined in a recent pro-con debate [36, 37]. Good compliance to CPAP, i.e. mean nightly use ≥ 4 hours, was associated with some protection, especially for occurrence of stroke [38].

Another area of current interest is whether OSA may modify the outcomes of acute coronary syndromes (ACS). OSA prevalence is high in patients with ACS, and severe OSA occurs in 25% of the patients [39]. The ongoing ISAACC trial (Impact of Continuous Positive Airway Pressure on Patients with ACS and Nonsleepy OSA) will provide long-term data on the effects of treating OSA in this population [40].

#### Arrhythmias

Arrhythmias are frequent in OSA patients, especially atrial fibrillation (AF). A permissive role of OSA towards the arrhythmogenic mechanism of AF is suggested by the higher risk of recurrence of AF in patients with OSA compared to non-OSA subjects and by the protective effect of CPAP treatment [41, 42]. Conversely, the literature on ventricular arrhythmias is relatively scarce and heterogeneous, as pointed out by a recent review [43]. Incidence of sudden cardiac death is increased at night in OSA according to some reports [44] while other studies found a uniform distribution over 24 hours [45]. Studies in patients with implantable cardioverter-defibrillator devices (ICD) have reported a high frequency of nocturnal discharge in OSA compared to non-OSA patients [46] or patients with chronic heart failure and central apneas [47, 48].

#### Cerebrovascular disease

Several studies reported an increased risk of stroke in snorers [49] and OSA patients [50]. CPAP treatment may reduce the risk of stroke [51], but most studies have used a composite cardiovascular outcome including stroke, rather than reporting data for each type of events. Available RCTs on the effects of CPAP in patients with stroke and OSA are usually short-term, and the low acceptance of CPAP treatment in patients with OSA and previous stroke is an additional difficulty to be considered. A recent meta-analysis on RCTs in the latter population reported improvement in neurological function in CPAP users [52]. More studies are necessary to evaluate the possible protective effects of CPAP on survival after stroke.

### Metabolic diseases

The relationship between OSA and metabolism is highly complex. On one hand, OSA is often associated with obesity, which by itself is characterized by disturbed energy metabolism and adipose tissue inflammation [53]. On the other hand, nocturnal intermittent hypoxia has been shown to affect glucose metabolism, and OSA could independently contribute to the pathogenesis of metabolic disorders [54]. The bidirectional relationships between OSA and disturbed energy metabolism [55] or type 2 diabetes [56] are current topics of interest, given the obesity epidemics and the increasing prevalence of type 2 diabetes worldwide.

#### OSA and the Metabolic Syndrome

The metabolic syndrome (MetS), a pre-diabetic state associated with central obesity and increased cardiovascular risk [57], is highly prevalent in OSA patients [58] and, according to some authors, OSA should be considered as an additional manifestation of MetS [59]. OSA may play a role in the pathogenesis of insulin resistance, the main feature of MetS, through intermittent hypoxia [60, 61] and sleep loss or fragmentation [62–64]. A fascinating recent research area is represented by the role of gut microbiota in metabolic derangements induced by intermittent hypoxia [65, 66] or sleep fragmentation [67]. Readers interested in the complex mechanisms of the interaction between OSA/intermittent hypoxia, adipocyte dysfunction, and inflammatory activation in adipose tissue, are referred to extensive reviews on these topics [68–71].

Although a positive effect of OSA treatment on metabolic disturbances might be expected based on the pathophysiological links described above, CPAP treatment does not modify visceral fat or metabolic variables [72, 73] unless concurrent weight loss occurs [74]. Nevertheless, short-term CPAP treatment for 8 hours/night improves insulin resistance, suggesting that prolonged nightly treatment with CPAP may be needed to modify glucose metabolism in OSA, possibly through decreased sympathetic activation [75]. Activity of insulin in the carotid body, and a common pathway involving both intermittent hypoxia and metabolism, is an interesting recent pathogenetic hypothesis possibly explaining the intertwining effects of OSA and glucose dysmetabolism [76].

#### OSA and diabetes

The bidirectional relationship between OSA and diabetes is especially interesting from a clinical point of view [77]. Treatment of OSA may help to prevent severe consequences of diabetes. This might indeed be the case, since although glycemic control does not improve during CPAP treatment according to meta-analyses [78, 79], untreated OSA in diabetic patients is associated with increased prevalence of neuropathy [80], peripheral arterial disease [81], diabetic retinopathy [82] and diabetic nephropathy [83–85]. Data on the effects of CPAP on diabetic complications are scarce. Compared to poorly compliant patients, optic nerve function improved in severe OSA patients with good compliance to CPAP treatment [86]. A recent *post-hoc* analysis of data from the SAVE study highlighted a higher risk of adverse outcomes in diabetic compared to non-diabetic patients, and a protective effect of CPAP on recurrent cardiovascular events only in diabetic patients with OSA showing a good adherence to CPAP treatment, i.e. at least 4 h/night, in the first 2 years of the study [87].

In summary, OSA may worsen metabolic abnormalities, and OSA treatment with sufficient adherence could play a protective role, especially when concomitant lifestyle interventions and weight loss are implemented. Screening for OSA in diabetic patients should be systematically done, since CPAP treatment for at least 4 h/night may be protective, especially when diabetic complications are also present [88].

### Renal disease

Renal diseases and OSA share common risk factors, like arterial hypertension, diabetes mellitus, obesity and advanced age. Each of such factors may give some independent contribution to the onset and progression of the other one [89]. OSA may endanger the kidney through several interacting mechanisms, including nocturnal intermittent hypoxemia, recurrent nocturnal blood pressure peaks, sympathetic hyperactivity, hyperactivation of intrarenal renin-angiotensin system, oxidative stress and systemic inflammation, endothelial dysfunction. A relationship between nocturnal hypoxemia and hyperactivation of the intrarenal renin-angiotensin system has been experimentally demonstrated [90].

Cross-sectional epidemiological studies have not consistently reported an association between OSA and either albumin excretion or eGFR. When an association was found, either severity of nocturnal hypoxemia [91] or the apnea/hypopnea index [92] were reported to correlate to renal alterations. These studies highly differed in design, sample size, recruitment criteria (patients referred to sleep laboratories, general population, diabetes as an inclusion or an exclusion criterion), so that it is difficult to draw firm conclusions from them.

More interesting and consistent results were obtained from longitudinal investigations. In a large study on US veterans, the annual rate of decline of eGFR was higher among patients diagnosed with sleep apnea than among controls [93]. Three retrospective cohort studies in Taiwan found a higher incidence of chronic kidney disease (any stage) among OSA than control subjects [94–96]. However, all these studies lacked polysomnographic information about OSA severity. Another longitudinal study on patients recruited in a sleep laboratory found that an accelerated decline in eGFR was more common among subjects who spent >12% of sleep time with oxygen saturation <90% than in less hypoxic subjects [97]. By contrast, a long-term study on the population-based Wisconsin Sleep Cohort did not find any difference in the rate of decline of eGFR between subjects initially showing an AHI>15 and other subjects [98]. However, the less severe nocturnal hypoxemia in sleep apnea subjects from the general population may at least partly explain the different results obtained in the Wisconsin cohort and in studies on OSA patients.

Most papers on the effects of OSA treatment on kidney function showed positive effects of CPAP. Two small studies on subjects with a high baseline GFR found a reduction of filtration fraction due to decrease in glomerular hyperfiltration [99, 100]. Two other small studies on subjects with mildly or severely impaired renal function observed an increase in eGFR [101] or a decrease in eGFR decline [102]. More recently, a RCT could not demonstrate a difference in the rate of eGFR decline between subjects with OSA and cardiovascular diseases treated by CPAP or under “usual care”; however, the power of the study could be insufficient to demonstrate a difference between the two groups [103]. In a study with a larger number of patients recruited in different sleep laboratories, therapy with fixed CPAP, but not with autoadjusting CPAP, could blunt the spontaneous trend of eGFR to decline over time [104].

In summary, there is some evidence that OSA may worsen kidney function through several mechanisms, and CPAP may exert beneficial effects.

### COPD

Both OSA and chronic obstructive pulmonary disease (COPD) are common and may occur in the same patient. Their association is known as “overlap syndrome” since the early studies [105]. Prevalence of the overlap syndrome has been reported at 1.0 to 3.6% in the general population, 8-56% in OSA patients, and 3-66% in COPD patients [106]. In OSA patients, prevalence of the overlap syndrome was found to increase with age, in agreement with COPD being more prevalent in elderly than middle-aged subjects [106]. In COPD patients, prevalence of respiratory events during sleep was high, with sleep disordered breathing (SDB) in 66% of patients with moderate to severe COPD [107]. In COPD patients undergoing pulmonary rehabilitation an AHI≥15/h was found in 45% of the sample [108]. COPD patients often show poor sleep quality [109, 110] and hypoventilation during sleep [111]. Use of oxygen during sleep could contribute to diagnostic uncertainty regarding OSA [107]. In addition, insufficient data are available on the role of either COPD or OSA severity on the clinical presentation or outcomes of the overlap syndrome, since the consequences of severe OSA associated with mild COPD may differ from those of mild OSA associated with severe COPD.

As outcomes are concerned, early studies reported lower PaO_2_ and higher PaCO_2_ in overlap patients compared to OSA patients with a similar AHI, associated with higher pulmonary artery pressure at rest and during exercise [112]. More recent observational studies reported increased mortality in overlap patients compared to OSA patients [113–115], and a protective effect of CPAP treatment [113, 116, 117]. By contrast, a complex study in over 6,000 subjects from the general population recently reported that mortality was higher in patients with SDB defined as AHI≥5, but occurrence of SDB and SDB severity could mitigate the effects of decreasing FEV_1_ on mortality [118]. These data suggest that OSA and COPD pathophysiology may interact, with low body mass index (BMI) and lung hyperinflation protecting against OSA in COPD, and upper airway and systemic inflammation in COPD potentiating the detrimental effects of OSA [119, 120]. Better phenotypic characterization of patients with overlap syndrome is needed, to optimize therapeutic strategies of both diseases.

### Asthma

Asthma and obstructive sleep apnea (OSA) are highly prevalent disorders which are often associated [121]. OSA symptoms are frequent in asthmatic patients [122–126], who also report daytime sleepiness [127], poor asthma control [128–131], and reduced quality of life [132]. Longitudinal data from the Wisconsin Sleep Cohort suggested that asthma at baseline increased the risk to develop OSA during follow up [133].

Sleep studies confirmed that OSA is more common in asthmatics than in controls [134–136], and OSA resulted associated with a higher frequency of asthma exacerbations [136]. Mild-moderate OSA occurred in 49% of patients with difficult-to-treat asthma [137]. Patients with severe asthma showed increased apnea-hypopnea index (AHI), poor sleep quality and daytime sleepiness [138]. However, lower airway resistance was shown to increase in asthmatic patients during slow wave sleep, whereas upper airway resistance remained low [139]. Moreover, hypopneas rather than apneas were the main type of respiratory events recorded in asthmatic patients [138]. On the other hand, upper airways in patients with OSA and asthma were shown to be smaller than in patients with either disease or controls, suggesting a synergistic role on upper airway inflammation played by both OSA and asthma [140].

In patients with suspected or confirmed OSA, some studies highlighted the association of asthma and obesity, especially in women [141–143]. In the European Sleep Apnea Database (ESADA), OSA and asthma were frequent in obese women [144]. A community-based study in Uppsala reported worse sleep quality and occurrence of nocturnal hypoxemia in women with both OSA and asthma, who showed higher BMI compared to controls or women with either asthma or OSA [145]. Other studies reported a positive relationship between severity of OSA and severity of asthma symptoms [138], higher prevalence of mild-moderate rather than severe OSA in patients with asthma [135, 143] or no relationship between asthma and OSA severity [6]. In the ESADA cohort, the distribution of OSA severity was similar in patients with and without physician-diagnosed asthma, and unaffected by treatment for asthma or for gastroesophageal reflux [144]. These differences in results among studies may at least partly reflect variable referral patterns for sleep studies in asthmatic patients, and further studies are needed to better define the real impact of OSA in asthma, and of asthma in OSA.

It is still uncertain whether treatment of OSA with continuous positive airway pressure (CPAP) might improve asthma control or pulmonary function. Some studies reported positive results [146–148] while other studies were negative [149, 150]. One study reported a decreased rate of FEV_1_ decline in asthmatic patients treated with CPAP [136], but the majority of studies agree on unchanged pulmonary function after CPAP. A recent systematic review pointed out that results of different studies do not allow to document a definite improvement in asthma control, although a positive effect of CPAP treatment seems to occur in patients with severe OSA or poorly controlled asthma [151].

In summary, the association of asthma and OSA would benefit from careful phenotyping of both diseases. Neutrophilic rather than eosinophilic inflammation was found in asthmatic patients with OSA [137, 152], suggesting a possible contribution of OSA to neutrophilic asthma. Further studies are needed to assess whether CPAP treatment could be a useful adjunct of asthma treatment in OSA patients, especially in cases of poorly controlled asthma.

### Cancer

The association of OSA and cancer has been explored in the last few years. In mice bearing human subcutaneous melanoma xenografts, intermittent hypoxia exposure accelerated tumor progression, and was associated with both metastases and resistance to treatment [153]. Such an effect was possibly mediated by activation of the hypoxia inducible factor (HIF) 1-alpha pathway [153, 154].

Epidemiological and clinical studies have explored the association of OSA and cancer in humans. Cancer mortality during follow up was increased in OSA patients compared to controls in general population samples [155, 156], cohorts of OSA patients [157], and cohorts of cancer patients [158], in association with OSA severity and duration of nocturnal hypoxemia. Some studies however did not show increased mortality associated with occurrence of OSA in the general population [159] or cohorts of cancer patients [160, 161].

Other studies reported increased incidence of cancer in cohorts of OSA patients compared to controls [156, 159, 162–165]; however, two population-based studies were negative [166, 167], but one study assessed only OSA symptoms rather than collecting objective sleep data [166]. According to some reports, incidence of cancer was especially high in relatively young OSA patients [157, 162]. Two studies assessing different cancer localizations reported a high risk of pancreatic cancer and melanoma in OSA patients, whereas risk for colorectal cancer was relatively low compared to non-OSA subjects [159, 160]. Therefore, although most studies indicate that intermittent hypoxia in OSA may increase cancer risk, firm evidence is still lacking, as confirmed by results of two recent meta-analyses [168, 169].

A series of studies focused on the association of OSA and cutaneous malignant melanoma (CMM), to verify whether data from the mouse model could be confirmed in humans. Tumor aggressiveness was increased in CMM patients with OSA and long time spent at low oxygen saturation (CT90%) or high oxygen desaturation index (ODI 4%) [158, 170–173]. Interestingly, tumor aggressiveness was positively associated with expression of the adhesion molecule VCAM-1 [171], HIF-1alpha [173], but not with expression of vascular endothelial growth factor (VEGF) [173]. Similar results were reported in patients with lung cancer and OSA [158].

In summary, the association of OSA and cancer is biologically plausible, as shown by the experimental studies using the intermittent hypoxia model. Human data on incidence of cancer and mortality in OSA patients confirm experimental data, especially in cohorts of CMM patients. However, no definitive evidence is available, and further studies are required especially concerning the possible higher risk of cancer in young OSA patients. Moreover, no study has assessed the potentially protective role of CPAP treatment, and studies based on administrative data often lack adjustments for known risk factors for cancer.

## Conclusions

Comorbidities are frequent in OSA patients, and OSA appears as a potential trigger for worse prognosis by worsening chronic organ damage [174], justifying the hypothesis of a dangerous liaison between OSA and comorbidities. Although the possible protective role of OSA treatment is still uncertain, it could differ among different clinical phenotypes of OSA patients. In that regard, studies are still moving their first steps [18, 175], but some data are available showing different responses depending on OSA phenotype [19]. Such view is confirmed by the recent report developed by European experts on OSA, which suggests that both symptoms and organ damage should be considered when choosing the appropriate treatment for OSA [174]. Although personalized medicine is slowly developing in the OSA field, testing a model similar to the model developed for COPD might provide useful hints on the possible detrimental role of comorbidities in OSA patients and suggest the best therapeutic approaches. Moreover, it is necessary to consider the role of comorbidities in elderly OSA patients and women with OSA, given the differences in pathophysiology and clinical presentation compared to the usual model of middle-aged men that dominates the current literature. Careful assessment of comorbidities should become standard clinical practice for OSA patients.

## Abbreviations

ACS: Acute Coronary Syndromes
AF: Atrial fibrillation
AHI: Apnea-Hypopnea Index
BMI: Body mass index
CMM: Cutaneous malignant melanoma
COPD: Chronic Obstructive Pulmonary Disease
CPAP: Continuous Positive Airway Pressure
eGFR: Estimated glomerular filtration rate
FEV_1_: Forced expiratory volume in 1 s
HIF: Hypoxia Inducible Factor
ICD: Implantable cardioverter-defibrillator
MetS: Metabolic syndrome
OSA: Obstructive sleep apnea
PaO_2_: Arterial partial pressure of oxygen
PaCO_2_: Arterial partial pressure of carbon dioxide
RCTs: Randomized controlled trials
SDB: Sleep Disordered Breathing
UA: Upper airways
VEGF: Endothelial Growth Factor
